# Supplementary material for: New Pyrimidine-5-Carbonitriles as COX-2 Inhibitors: Design, Synthesis, Anticancer Screening, Molecular Docking, and In Silico ADME Profile Studies
Source: Molecules. 2022 Nov 2;27(21):7485. doi: 10.3390/molecules27217485 (PMC9658812; doi:10.3390/molecules27217485)
Supplement: Supplementary file 1 [file molecules-27-07485-s001.zip › molecules-1987639-supplementary.pdf]

New pyrimidine-5-carbonitriles as COX-2 inhibitors: Design, synthesis, anticancer screening, molecular docking, and in silico ADME profile studies

Hanan A. AL-Ghulikah[1], Samiha A. El-Sebaey[2*], Amr K. A. Bass [3], Mona S. El-Zoghbi [3]

[1]Department of Chemistry, College of Sciences, Princess Nourah bint Abdulrahman University, P.O. Box 84428, Riyadh 11671, Saudi Arabia

[2] Department of Pharmaceutical Organic Chemistry, Faculty of Pharmacy (Girls), Al-Azhar University, Youssef Abbas Street, Nasr City, Cairo, Egypt

[3] Department of Pharmaceutical Chemistry, Faculty of Pharmacy, Menoufia University, Menoufia, Shibin-Elkom, Gamal Abd Al-Nasir Street, Egypt

Corresponding author mail: samiha.ali85@azhar.edu.eg

S1

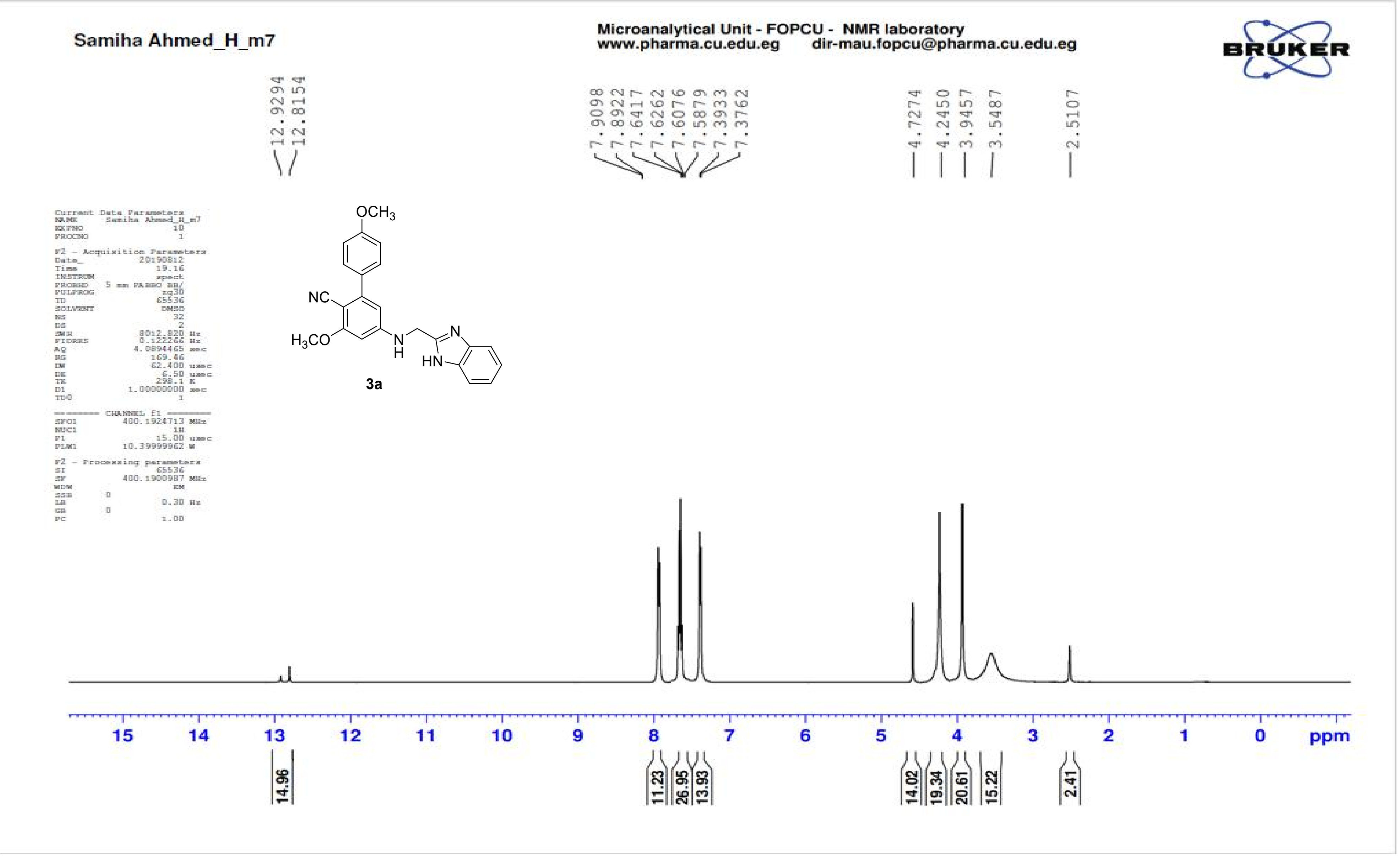

Figure S1. ^{1}H NMR of compound **3a**.

S2

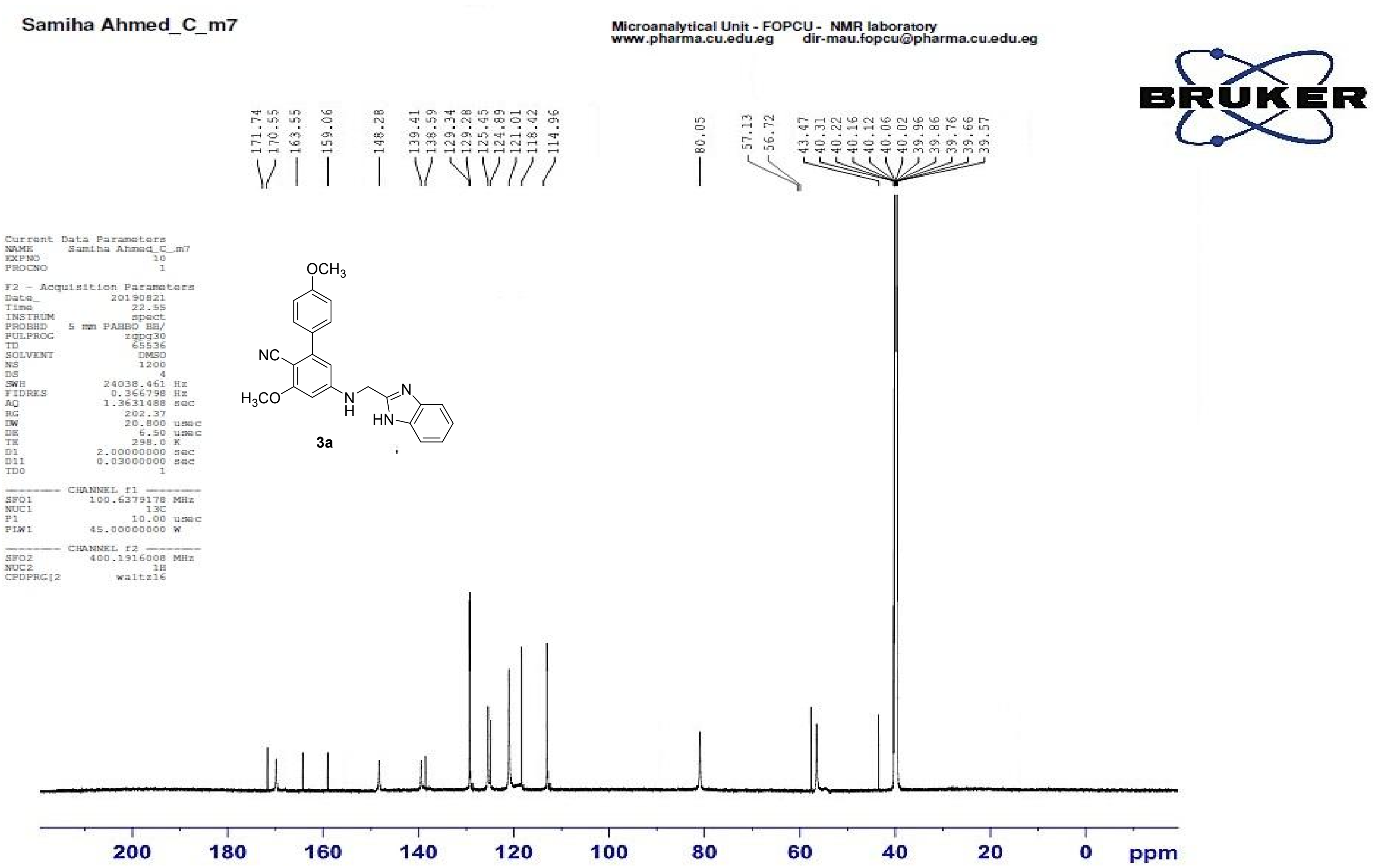

Figure S2. ^{13}C NMR of compound **3a**.

S3

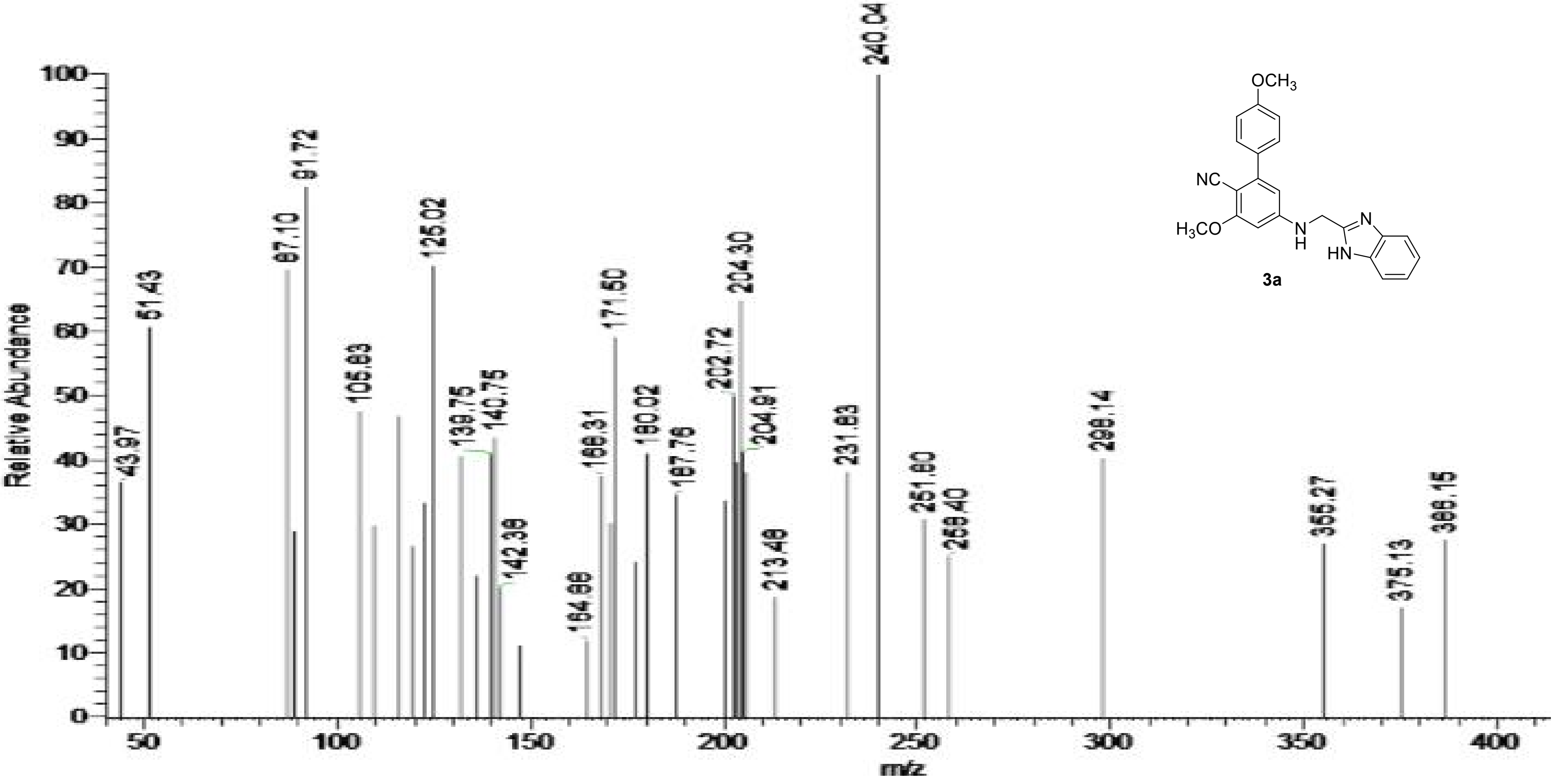

Figure S3. Mass of compound **3a**.

S4

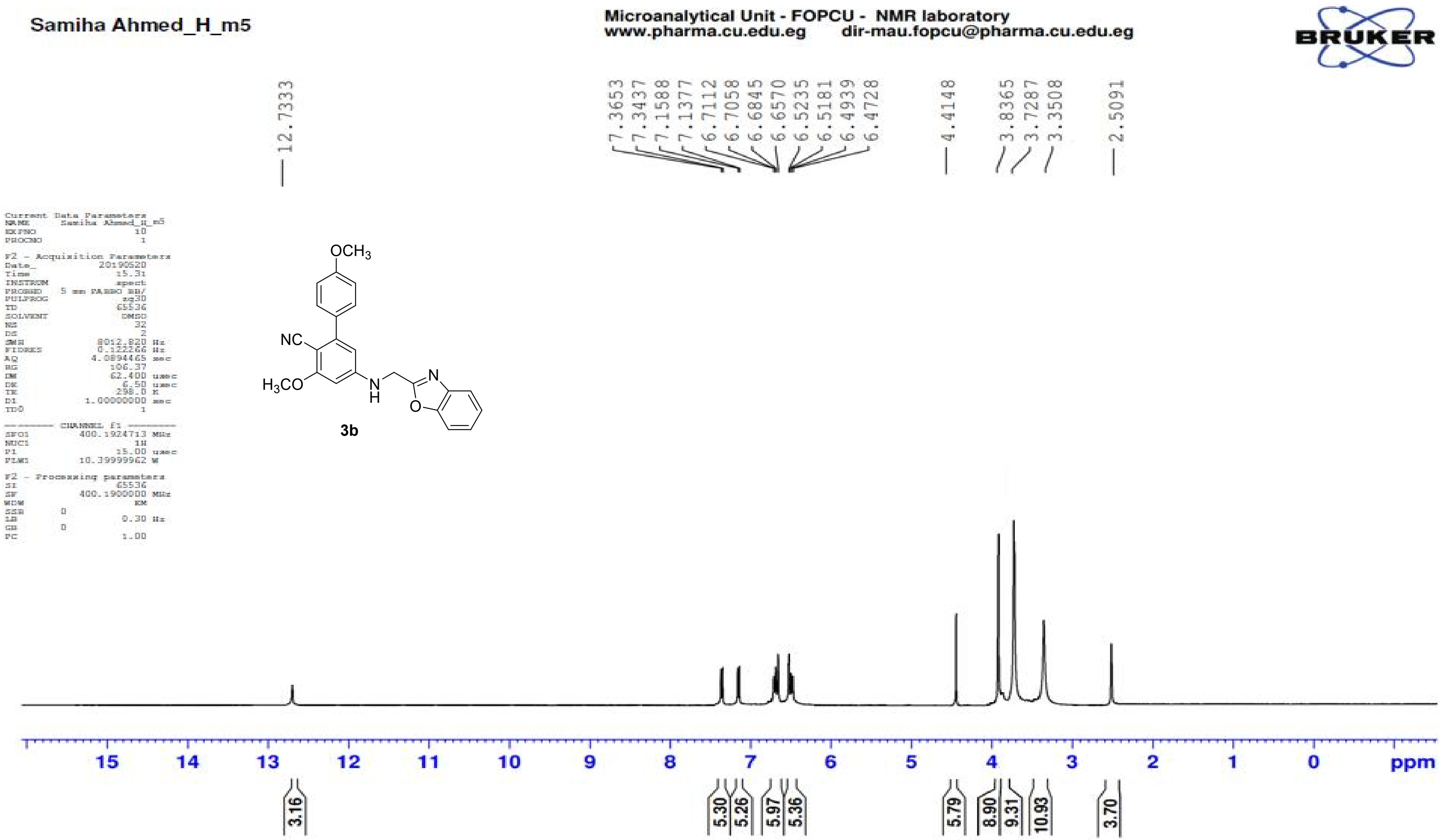

Figure S4. ^{1}H NMR of compound **3b**.

S5

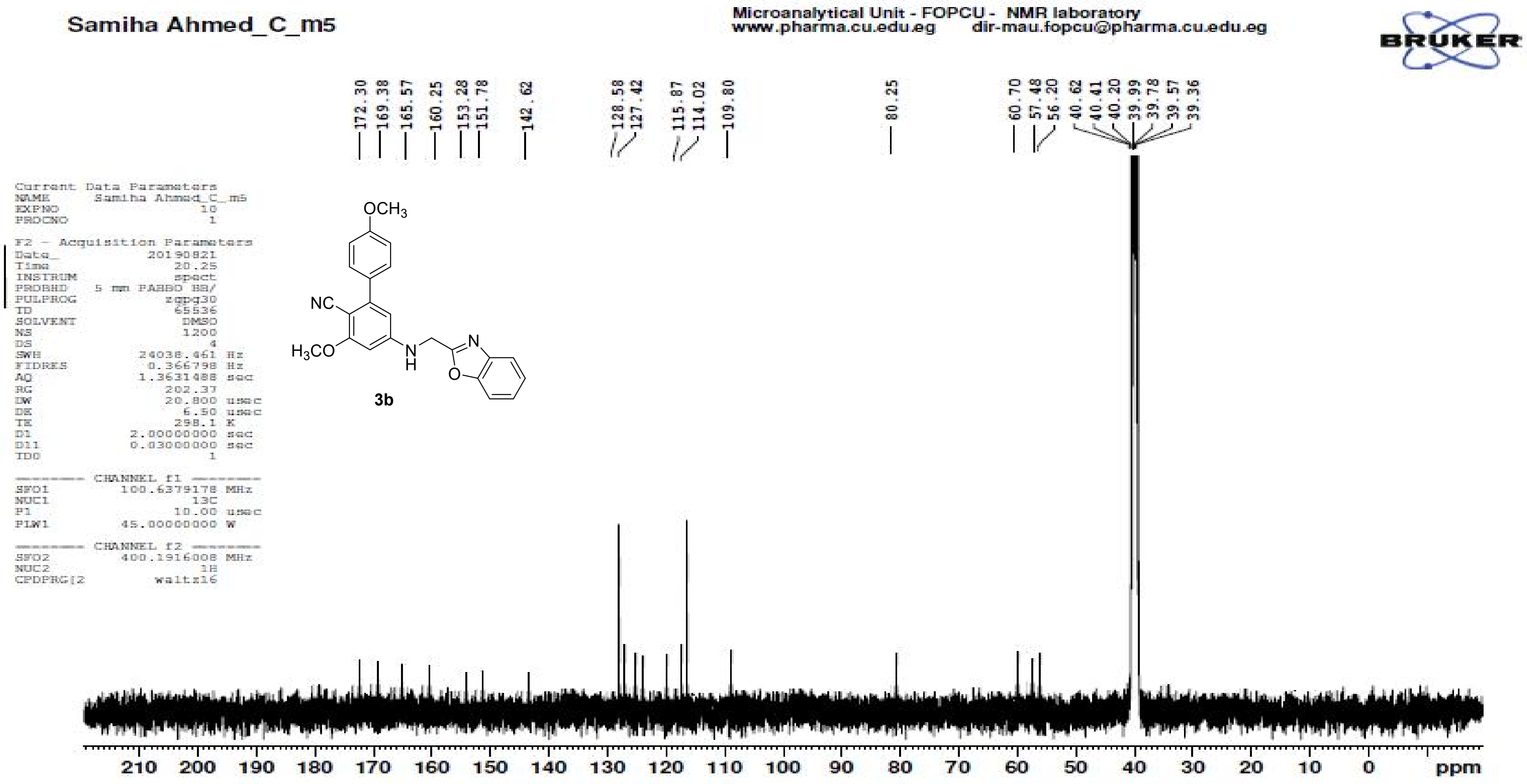

Figure S5. ^{13}C NMR of compound **3b**.

S6

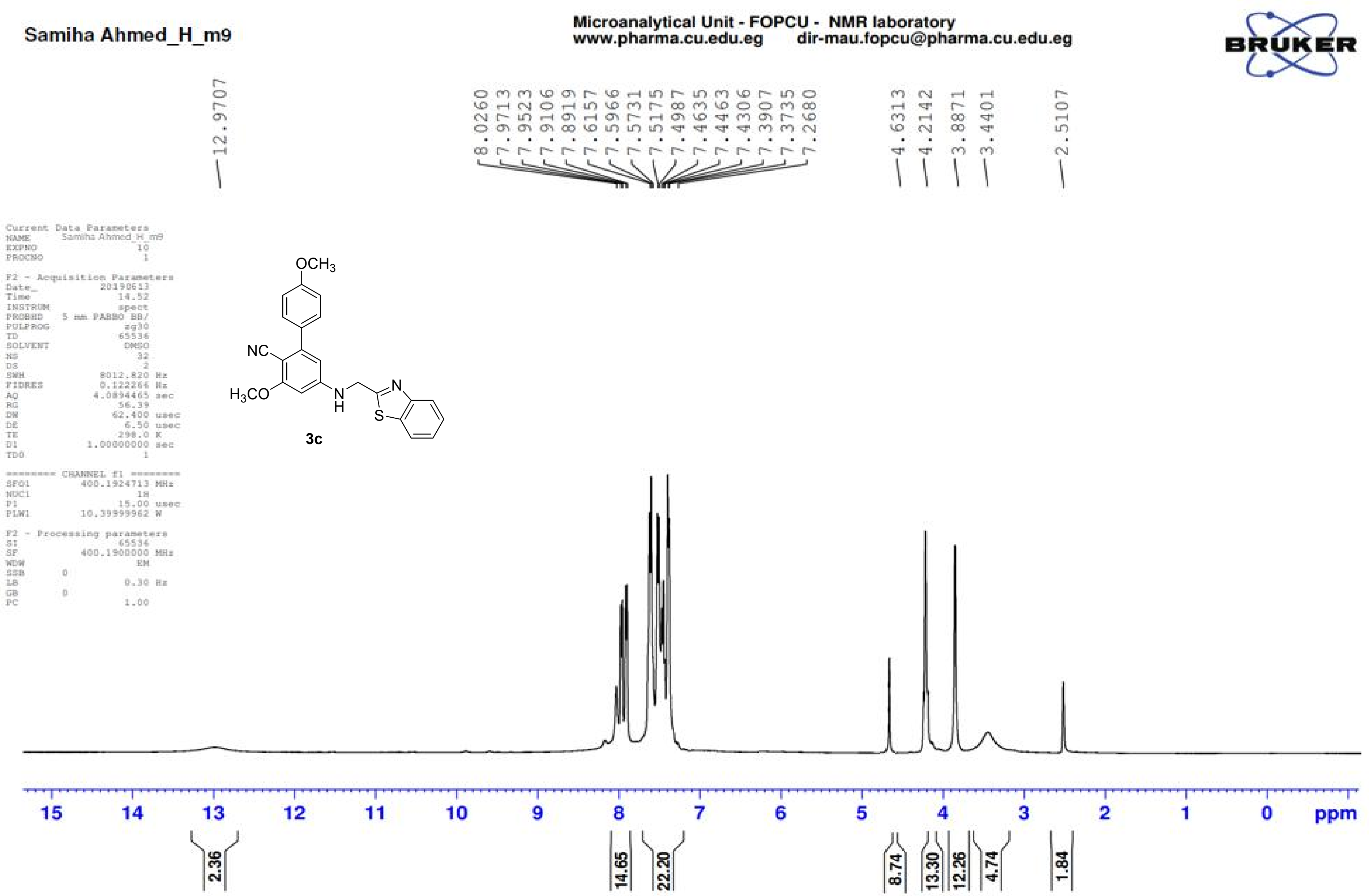

Figure S6. ^{1}H NMR of compound **3c.**

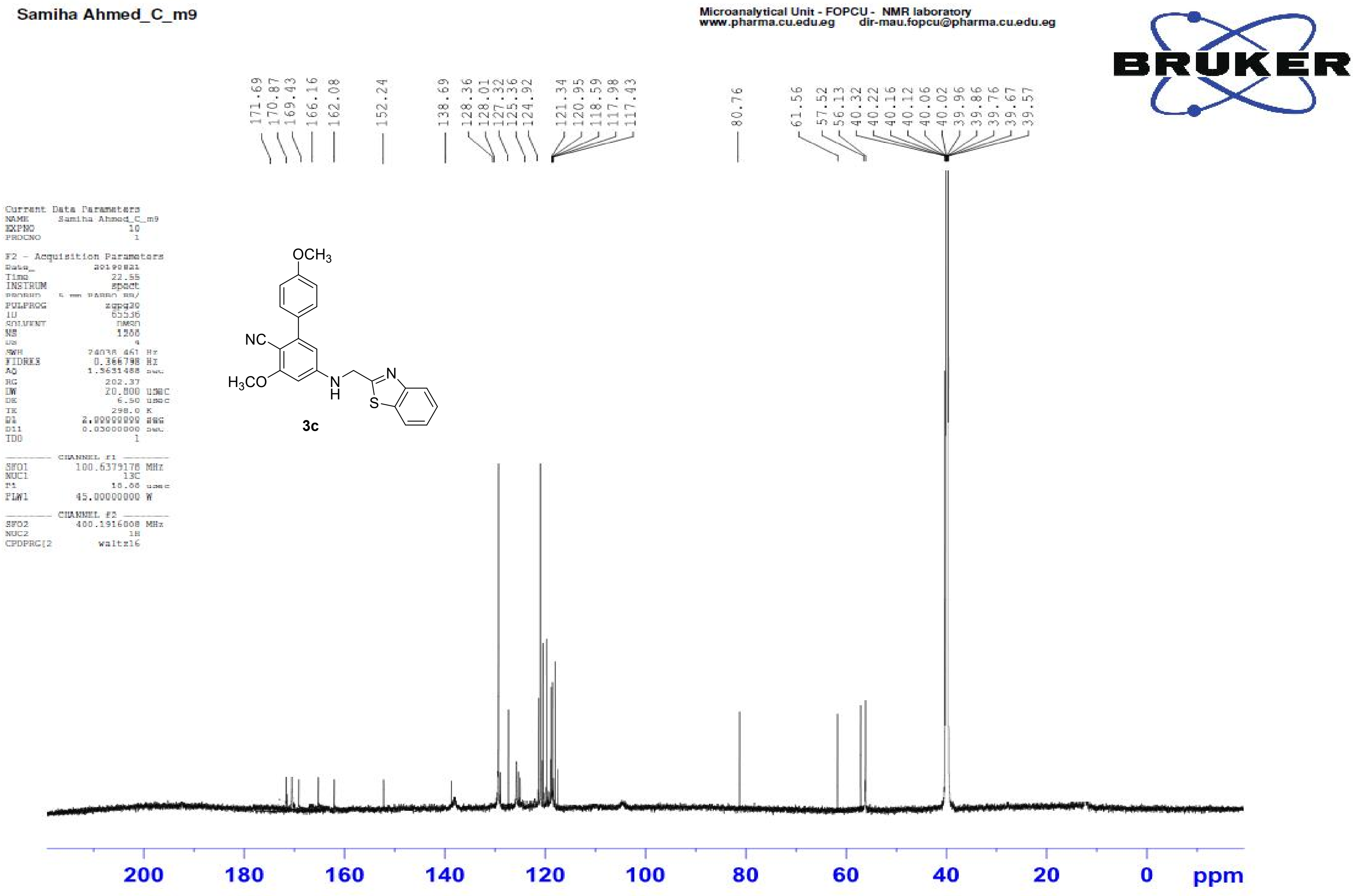

Figure S7. ^{13}C NMR of compound **3c**.

S8

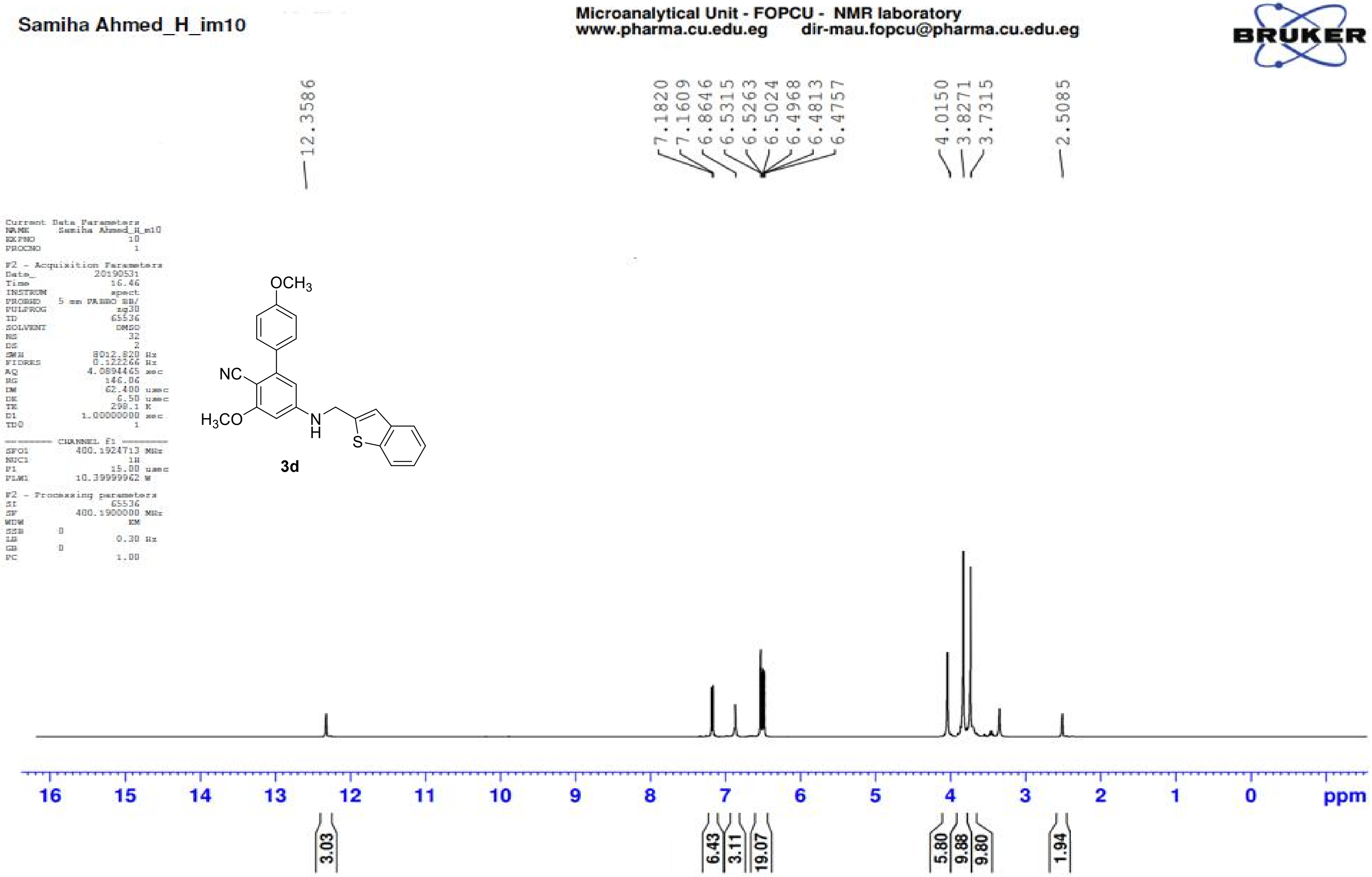

Figure S8. ^{1}H NMR of compound **3d**.

S9

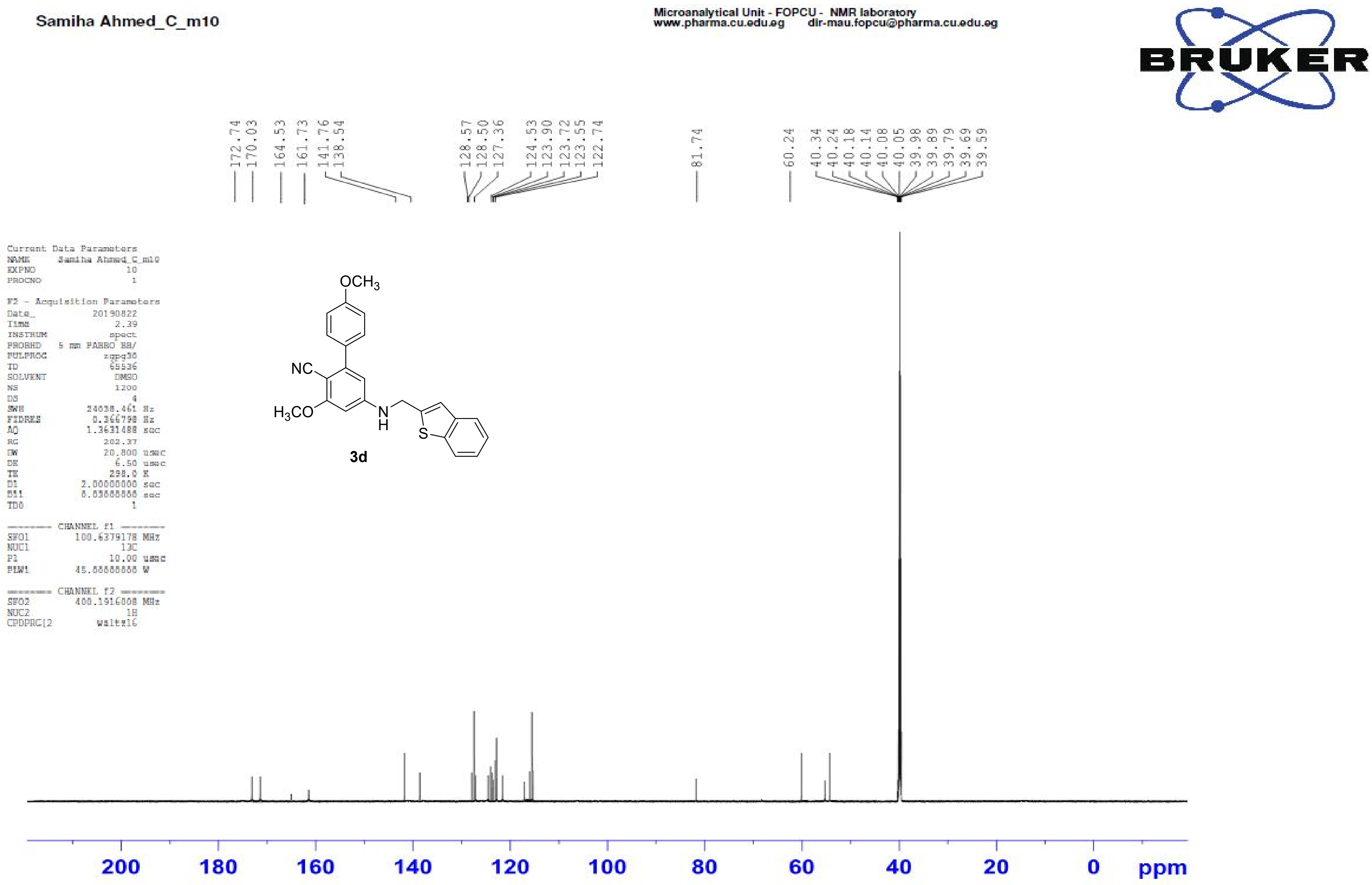

Figure S9 .^{13}C NMR of compound **3d**.

S10

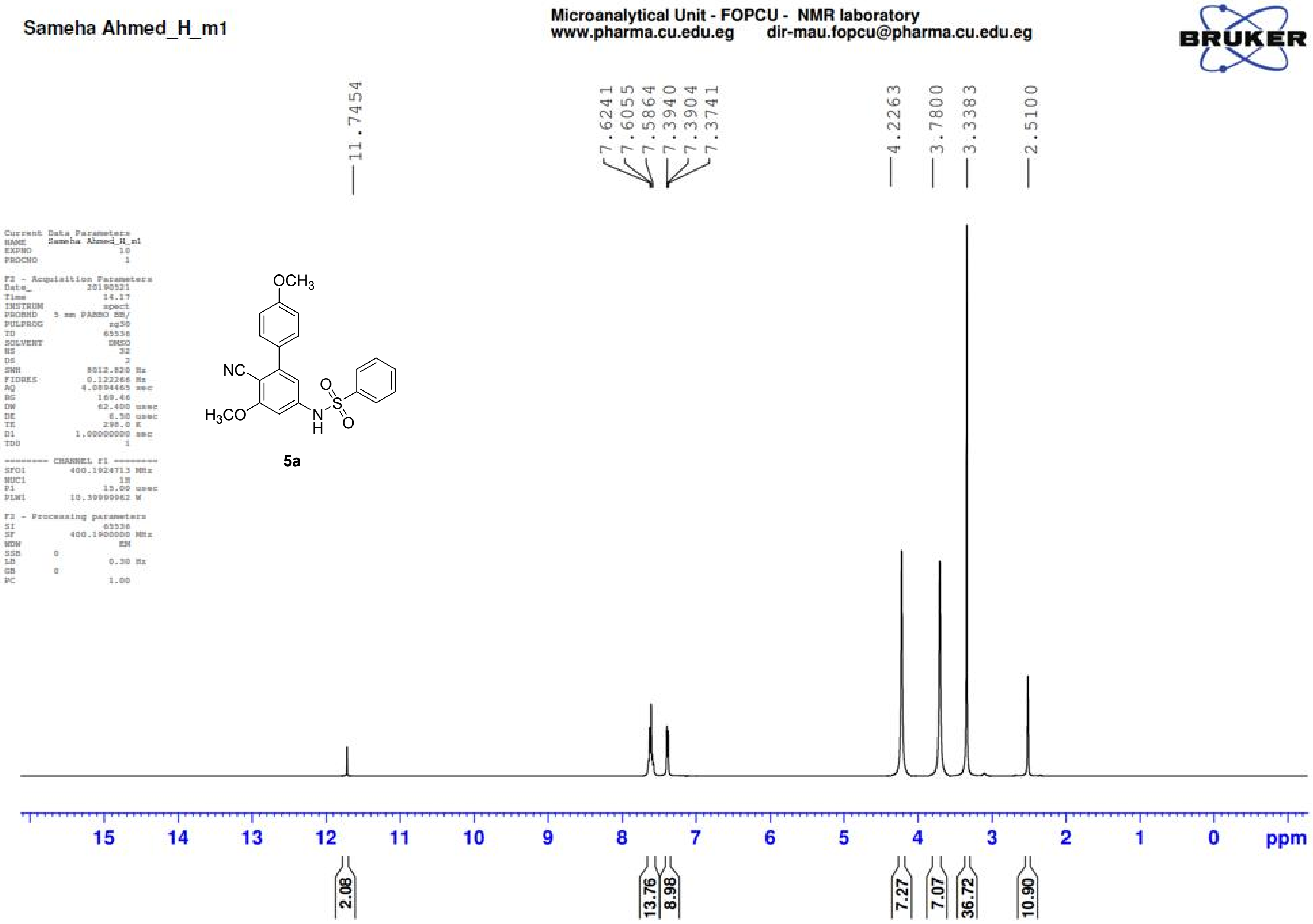

Figure S10. ^{1}H NMR of compound **5a**.

S11

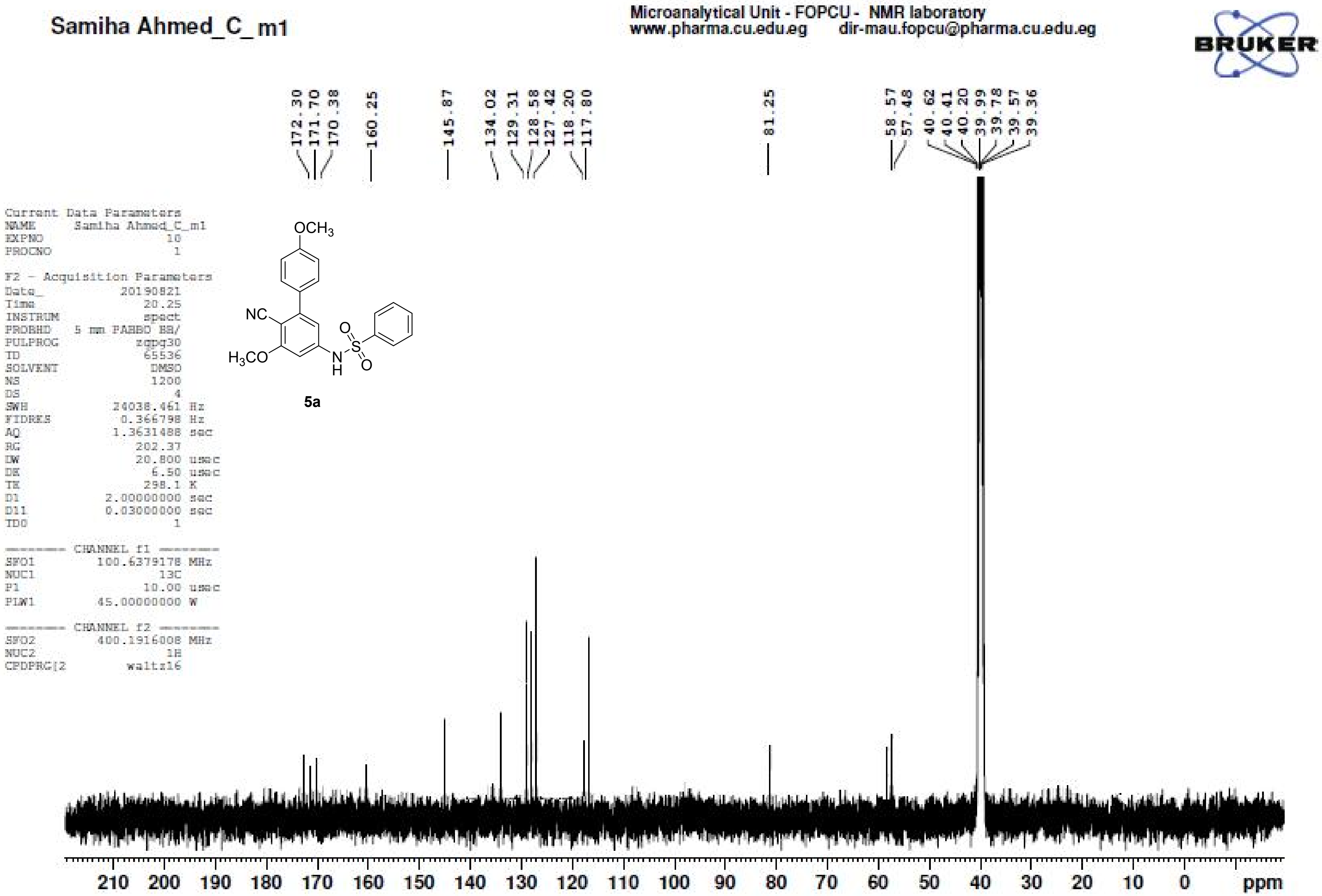

Figure S11. ^{13}C NMR of compound **5a.**

S12

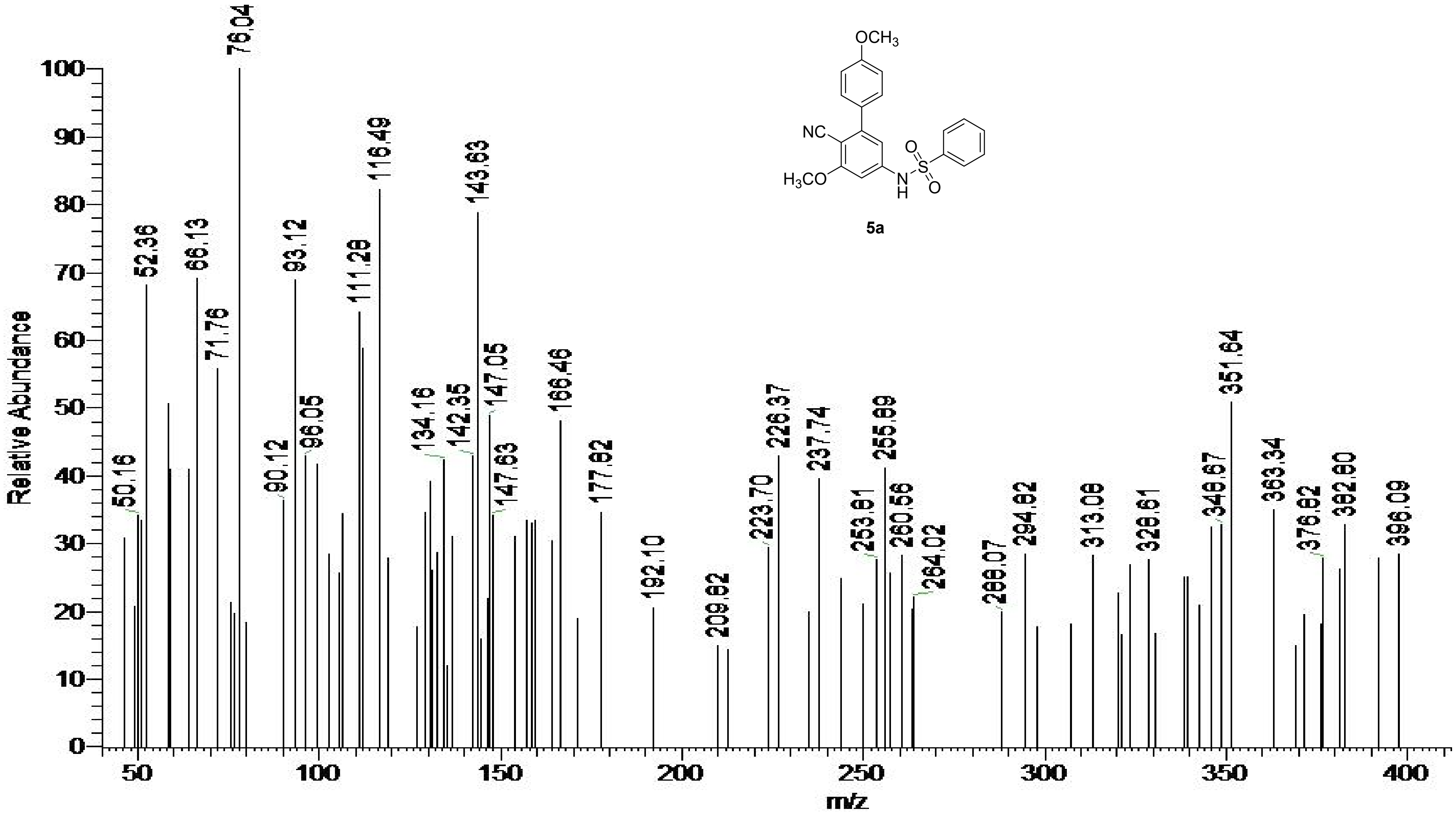

Figure S12. Mass of compound **5a.**

Samiha Ahmed_H_m4

Microanalytical Unit - FOPCU - NMR laboratory
www.pharma.cu.edu.eg dir-mau.fopcu@pharma.cu.edu.eg

S13

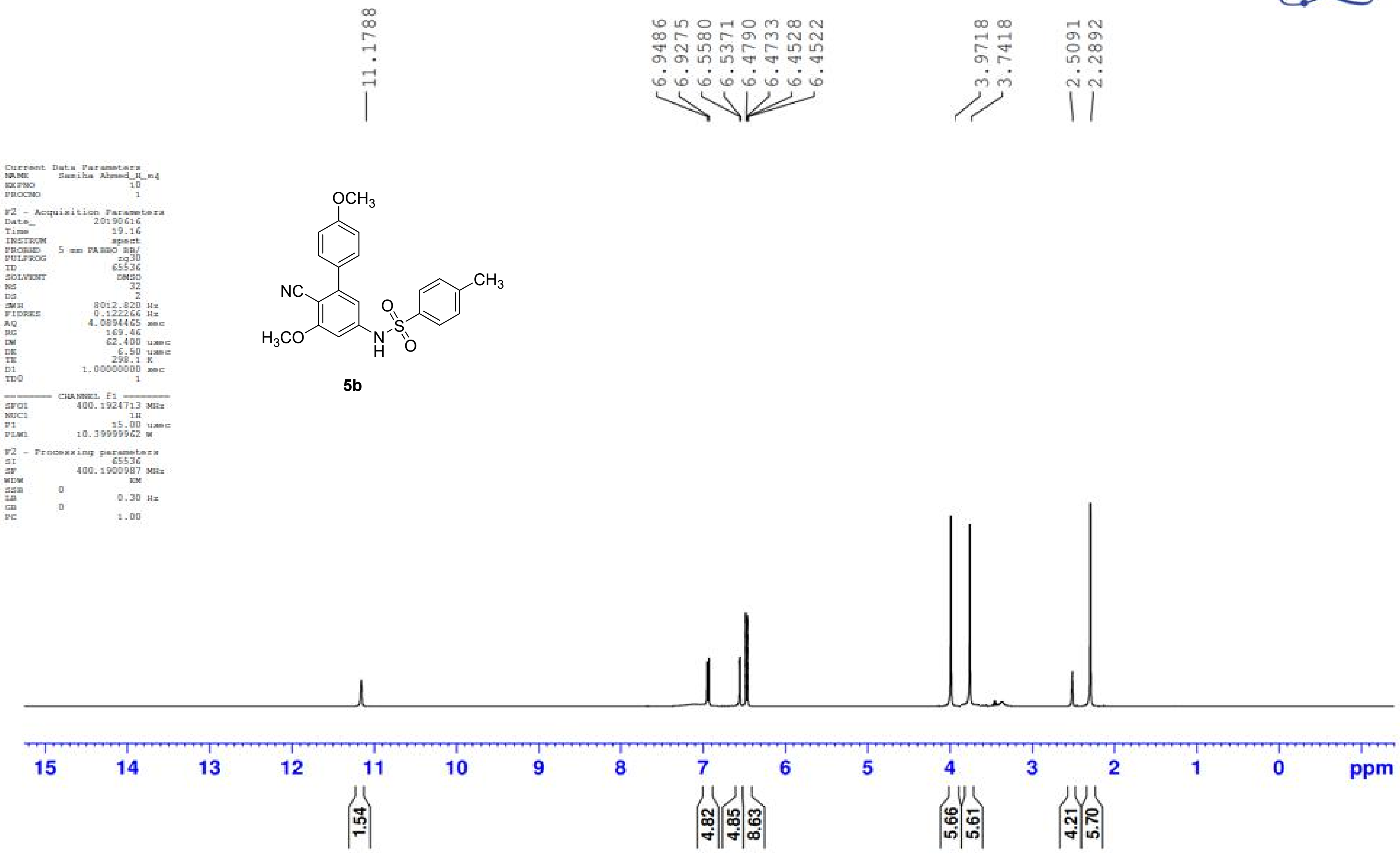

Figure S13. ^{1}H NMR of compound **5b**.

S14

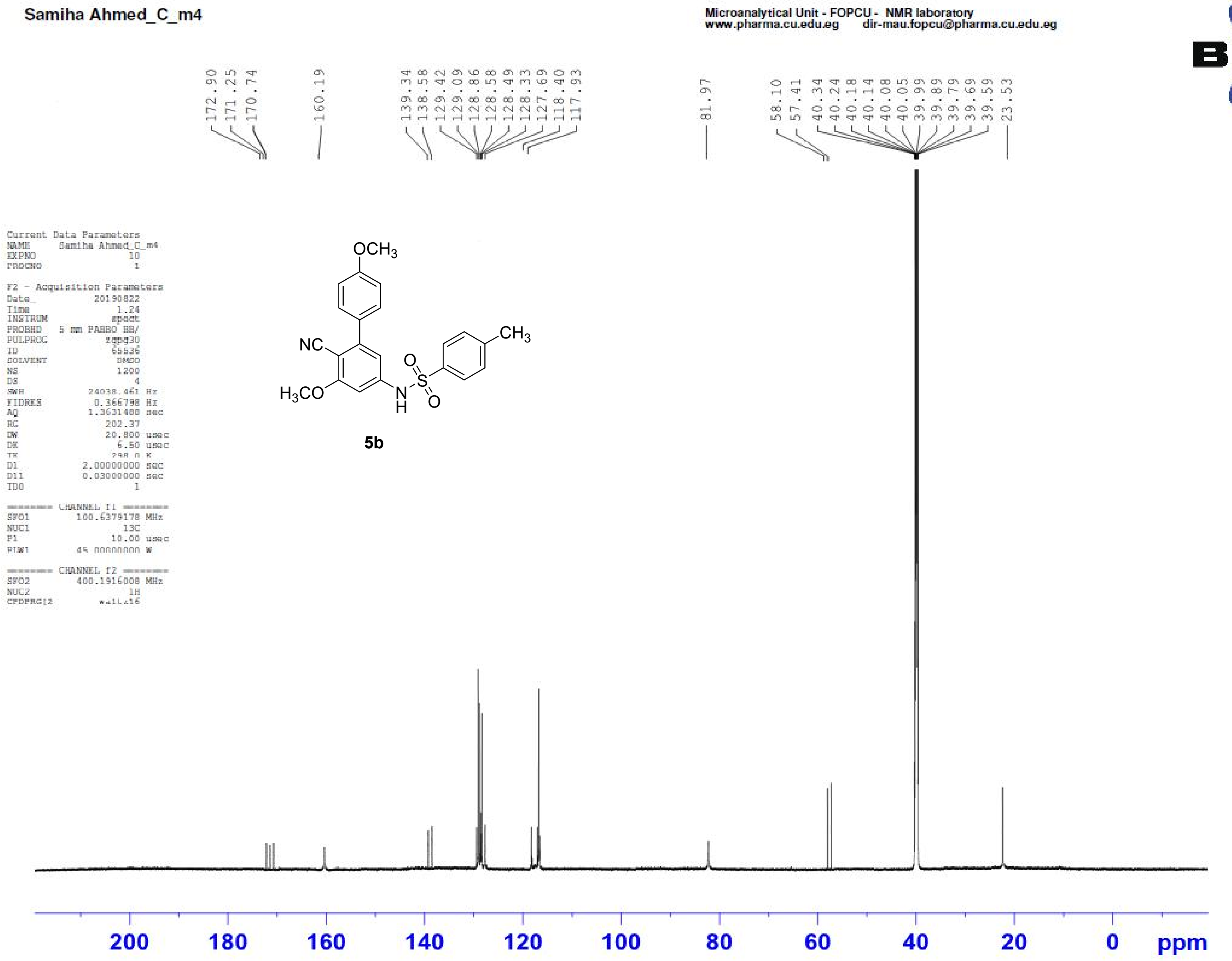

Figure S14. ^{13}C NMR of compound **5b**.

S15

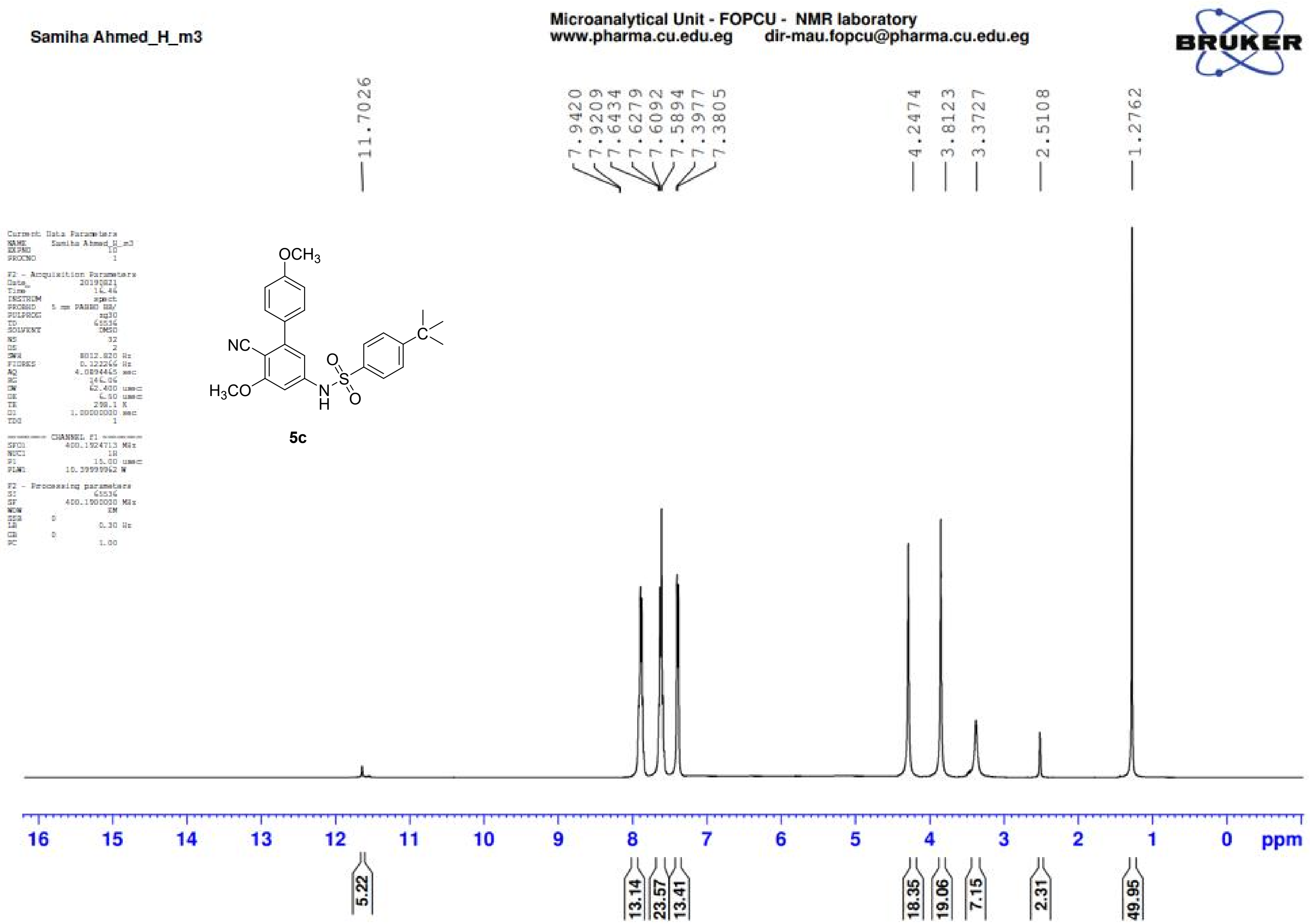

Figure S15. ^{1}H NMR of compound **5c**.

S16

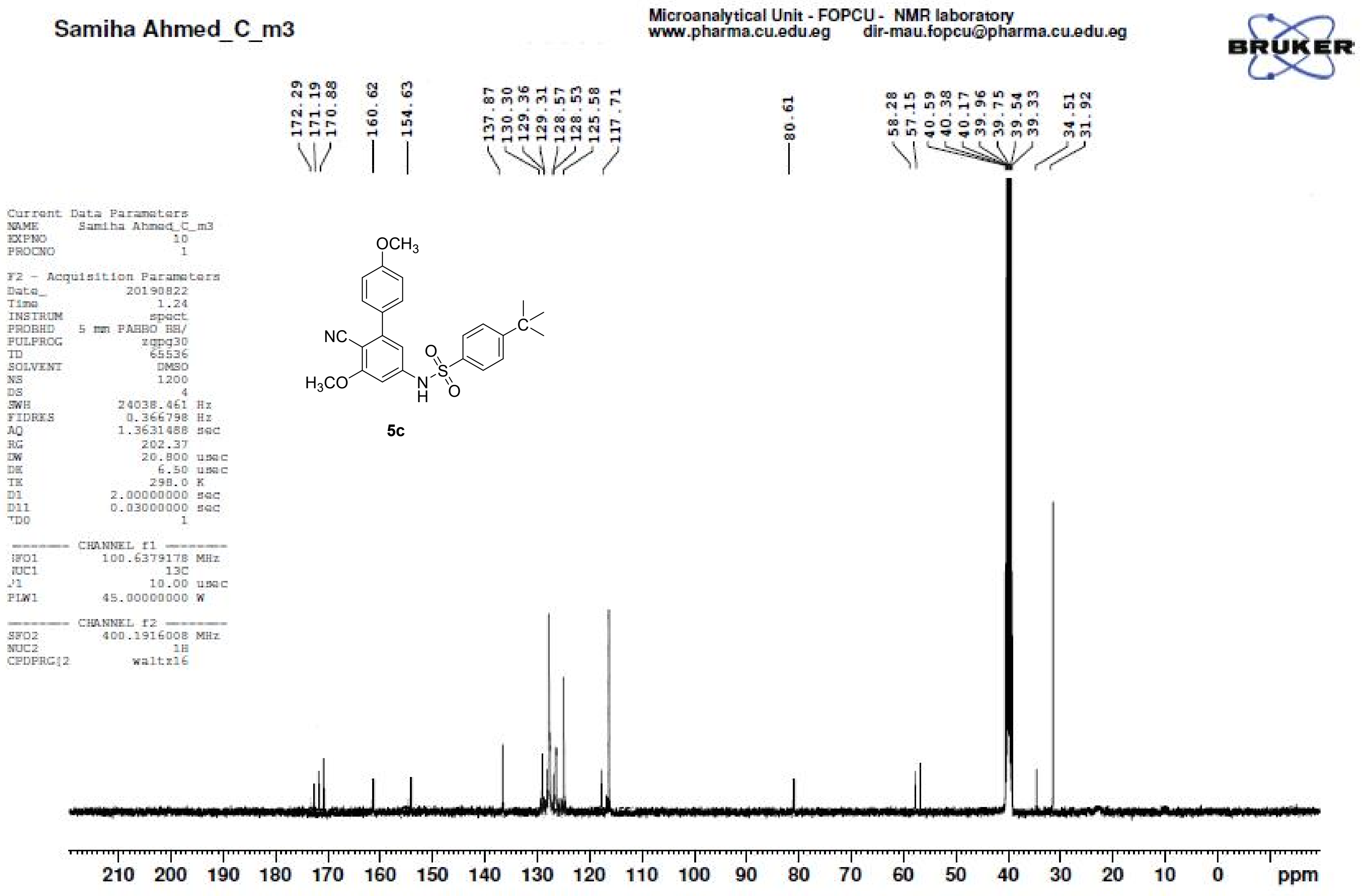

Figure S16. ^{13}C NMR of compound **5c**.

S17

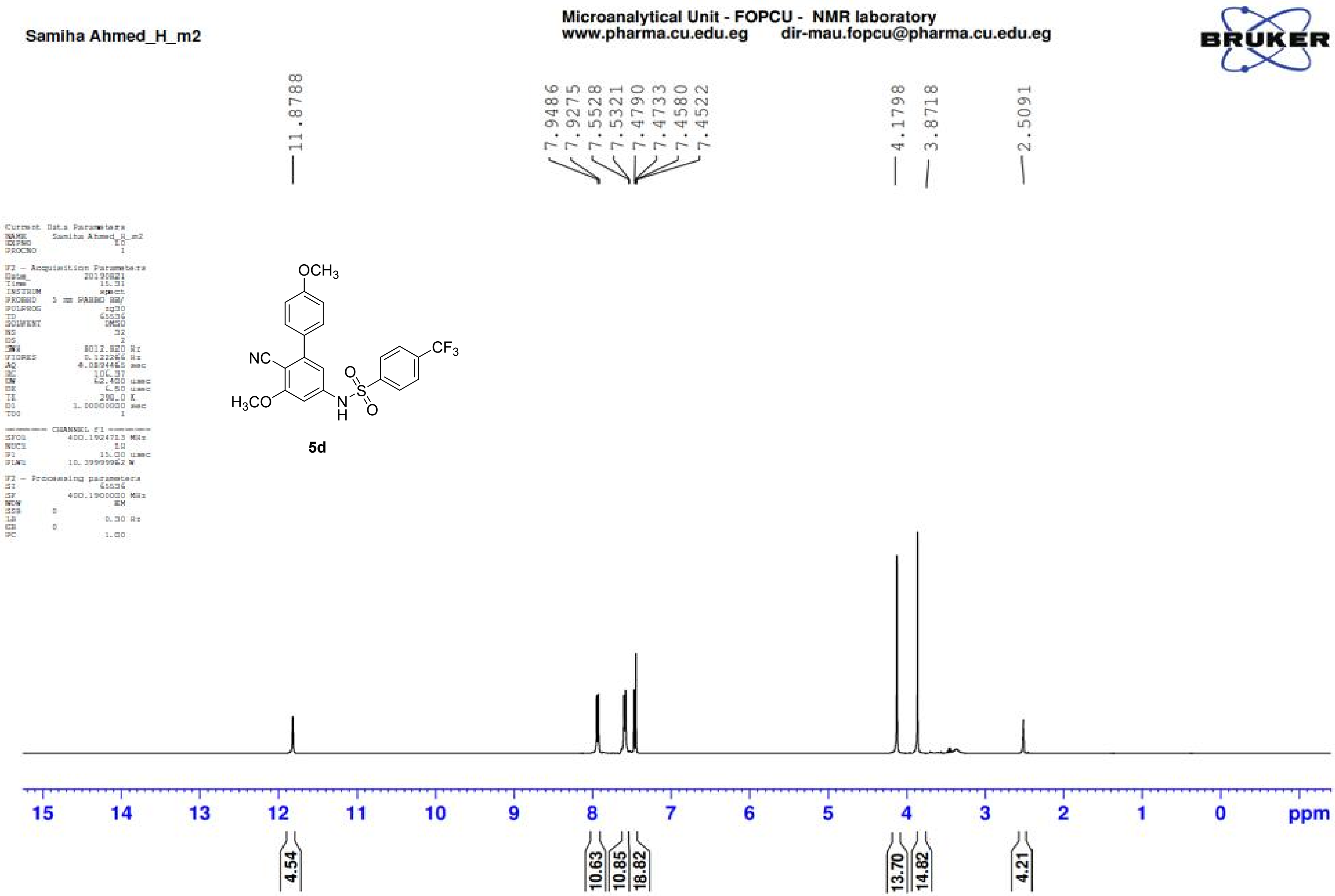

Figure S17. ^{1}H NMR of compound **5d**.

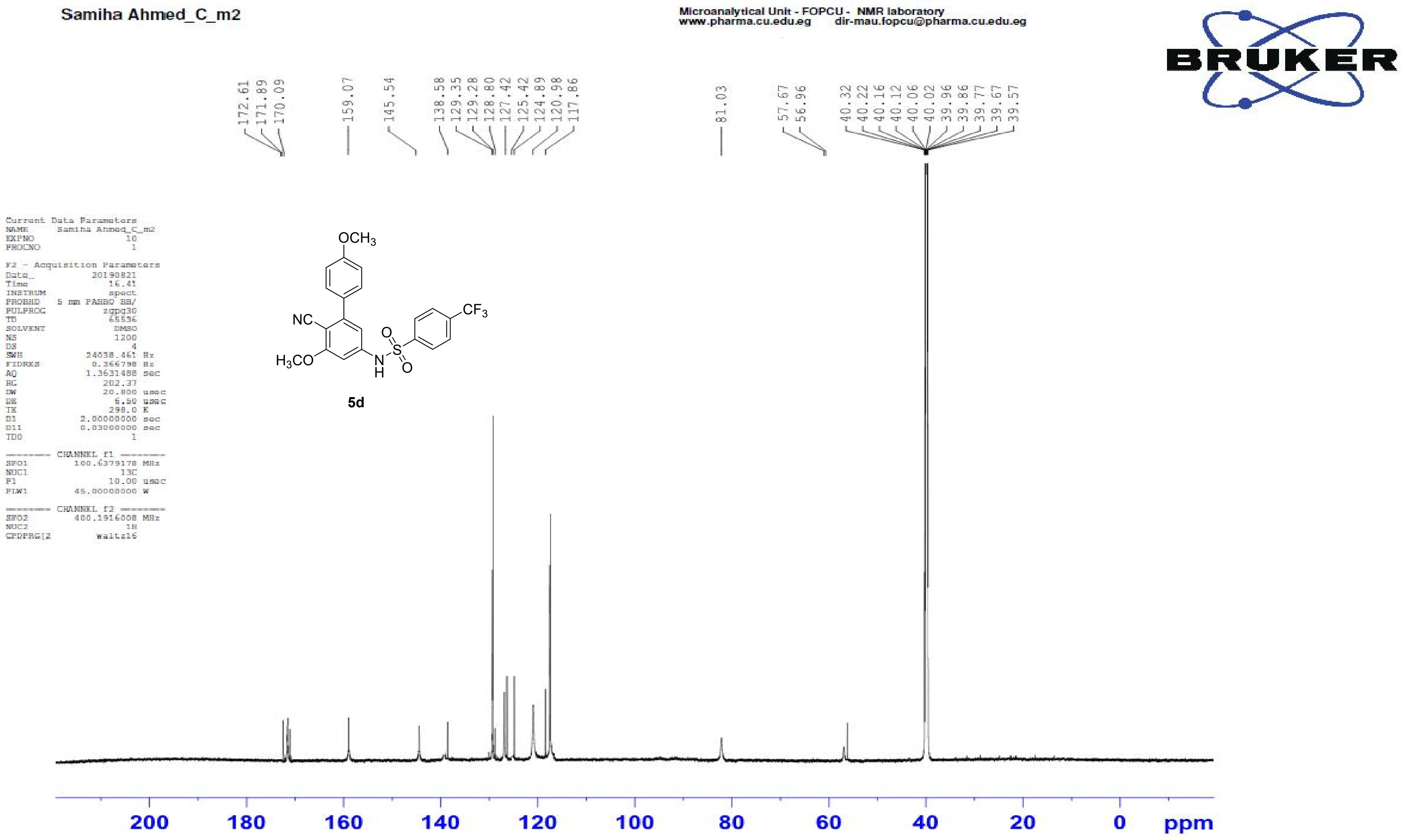

Figure S18. ^{13}C NMR of compound **5d**.
